# Long-Term Relief of Painful Bladder Syndrome by High-Intensity, Low-Frequency Repetitive Transcranial Magnetic Stimulation of the Right and Left Dorsolateral Prefrontal Cortices

**DOI:** 10.3389/fnins.2018.00925

**Published:** 2018-12-11

**Authors:** Julien Nizard, Julien Esnault, Bénédicte Bouche, Alcira Suarez Moreno, Jean-Pascal Lefaucheur, Jean-Paul Nguyen

**Affiliations:** ^1^Multidisciplinary Pain, Palliative and Support Care Center, UIC22, PHU2 and EA3826, University Hospital Nantes, Nantes, France; ^2^Multidisciplinary Pain Center, Clinique Brtch, Groupe ELSAN, Nantes, France; ^3^Clinical Neurophysiology Department and EA4391, Henri Mondor University Hospital, and UPEC Faculty of Medicine, Crteil, France

**Keywords:** bladder pain syndrome, central sensitization, dorsolateral prefrontal cortex, interstitial cystitis, neuromodulation, transcranial magnetic stimulation

## Abstract

**Aim:** To show the value of low-frequency repetitive transcranial magnetic stimulation (rTMS) of the dorsolateral prefrontal cortex (DLPFC) to treat bladder pain syndrome (BPS), characterized by suprapubic pain, urgency and increased micturition frequency.

**Methods:** A 68-year-old woman with BPS underwent 16 sessions of high-intensity, low-frequency (1 Hz) rTMS of the DLPFC, first on the right hemisphere (one daily session for 5 days, followed by one weekly session for 5 weeks), and then on the left hemisphere (one monthly session for 6 months).

**Results:** At the end of the rTMS protocol, suprapubic pain completely vanished, micturition frequency dramatically decreased (by 60–80%), while fatigue and sleep quality improved (by 57–60%). The patient reported an overall satisfaction rate of 80% and her activities of daily living tending to normalize.

**Conclusion:** This is the first report showing that high-intensity, low-frequency rTMS delivered on the DLPFC region of both hemispheres can relieve most symptoms of BPS (pain, urinary symptoms, and interference with physical functioning) in clinical practice.

## Introduction

Bladder pain syndrome (BPS), also known as “interstitial cystitis,” is pain a relatively common disease, with a prevalence of about 2.5%, affecting women about four times more than men (van de Merwe et al., 2008; Offiah et al., 2013; Hanno et al., 2015). BPS is mainly characterized by disabling suprapubic pain, urge to urinate, and increased micturition frequency (van de Merwe et al., 2008; Offiah et al., 2013; Hanno et al., 2015). The etiology of BPS is unknown, but symptoms are thought to arise from chronic inflammation of the bladder wall in the absence of infection. The involved pathophysiological mechanisms include urothelial permeability alteration, impaired glycosaminoglycan layer in the bladder, abnormal mast cell activation, or neuroendocrine changes (Patnaik et al., 2017). However, chronic inflammation is mostly associated with the release of various factors and cytokines that may contribute to induce a hyperexcitation of nerve fiber endings in the bladder wall, and then a central sensitization of ascending pathways and brain structures involved in sensory information integration (Yoshimura et al., 2014a,b). Misperceptions and improper integration of sensory information provided by bladder filling may be responsible for urinary problems and bladder pain. A phenomenon of central sensitization triggered by peripheral inflammation also characterizes other “dysfunctional” chronic pain syndromes (Latremoliere and Woolf, 2009), such as glossodynia, temporomandibular joint syndrome, fibromyalgia, and irritable bowel syndrome, which may be associated with BPS (Alagiri et al., 1997). Neuromodulation techniques can act on central pain sensitization by activating brain structures that control the integration of afferent nociceptive information. For this purpose, it has been proposed to use repetitive transcranial magnetic stimulation (rTMS) delivered at high “excitatory” frequency (≥5 Hz) to treat chronic pain syndromes (Lefaucheur et al., 2011; Nizard et al., 2012).

In addition to “ascending” controls, central sensitization also includes “descending” controls, such as descending facilitatory projections from the rostral ventromedial medulla (RVM) (Burgess et al., 2002; Staud, 2013). In contrast to high-frequency rTMS, low-frequency (1 Hz) rTMS rather produces “inhibitory” effects and if such an inhibitory stimulation is applied to certain cortical regions connected to the RVM, the effect of the descending sensitization could be blocked at the level of this medullary structure. One of these cortical regions is the dorsolateral prefrontal cortex (DLPFC), which is readily accessible to rTMS. In the domain of therapeutic applications of rTMS, low-frequency (1 Hz) rTMS of the right DLPFC is a procedure that has shown its efficacy in the treatment of depression or fatigue (Lefaucheur et al., 2014, 2017).

In a patient with BPS refractory to usual therapeutic approaches and suffering from the coexistence of pain and depressive symptoms, we proposed rTMS therapy. Unfortunately, this patient did not improve after high-frequency (10 Hz) rTMS delivered to the left or right motor cortex (the usual target for treating pain, Lefaucheur et al., 2014) or the left DLFPC (the usual procedure for treating depression, Lefaucheur et al., 2014). On the other hand, he clearly responded to low-frequency (1 Hz) rTMS of the right DLPFC, especially regarding the intensity of suprapubic pain and the number of micturitions, which were reduced by 30–40%. Despite this improvement, the patient considered that he remained significantly impaired by the persistence of a significant nocturia and decided to stop this treatment. However, this first experience showed that 1 Hz rTMS of the right DLPFC could be beneficial in patients with BPS. Therefore, we applied this procedure to a second patient. She clearly benefited from repeated sessions of 1 Hz rTMS of the right DLPFC, especially concerning pain and the number of micturitions. We report this original clinical experience.

## Case Report

**FIGURE 1:**
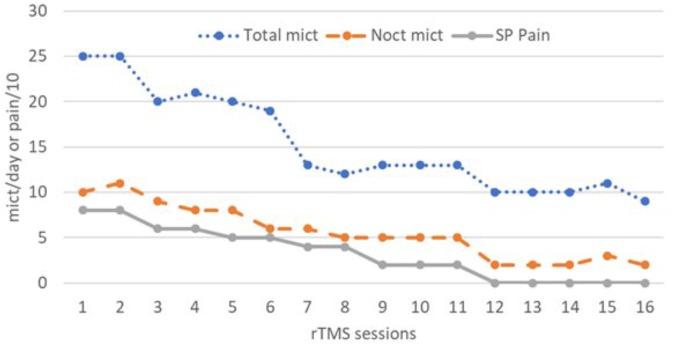
Total daily number of micturitions (Total mict), number of nocturnal micturitions (Noct mict), and suprapubic pain intensity rated on a 0–10 numeric scale (SP Pain), after 16 sessions of 1 Hz repetitive transcranial magnetic stimulation (rTMS) delivered to the right (sessions 1 to 10) or left (sessions 11 to 16) dorsolateral prefrontal cortex (DLPFC).

A 68-year-old woman presented features of BPS since 2014. She had undergone hysterectomy in 1993, which was followed by genital prolapse. She subsequently developed perineal pain without urinary symptom, which was attributed to pudendal neuralgia. Surgical decompression of the right pudendal nerve was performed in 2000. However, perineal pain significantly persisted, and gradually acquired neuropathic features. Spinal cord stimulation was initiated in 2005, with good efficacy [mean reduction of perineal pain scores from 8 to 2/10 on a numerical rating scale (NRS) from 0 to 10] and the stimulator is still activated until now with the same efficacy on this type of perineal pain. However, in 2014, the patient developed a totally new painful syndrome, characterized by suprapubic pain (NRS: 8/10) associated with urgency and increased micturition frequency with an average of 25 micturitions per 24 h, including 10 nocturnal micturitions. Such symptoms, in the absence of urinary tract infection or other cause of lower urinary tract dysfunction, defined BPS, although cystoscopy did not show Hunner ulcer lesions. In detail, the pain syndrome presented by the patient included a permanent pain described as a sharp and burning sensation, limited to the suprapubic region, and paroxysms of increasing pain described as a sensation of pressure, more diffuse in the hypogastric region and leading to uncomfortable urge to urinate. The clinical picture was also characterized by mood disorder (HAD, Hospital Anxiety and Depression scale: 24/42), sleep disturbance (NRS: 7/10), and fatigue (NRS: 5/10) with a major impact on activities of daily living. Cystoscopy and bacteriological urine examination excluded urinary tract infection. Bladder hydrodistention was performed but was not beneficial. Treatment with pentosan polysulfate was also ineffective. An rTMS therapy was then proposed to the patient. According to the results obtained in a previous patient with BPS (see above), we performed high-intensity, low-frequency rTMS of the right DLPFC (1 Hz, 1,200 pulses per session, delivered at 110% of the motor threshold, MT). The patient underwent one session per day for five consecutive days, followed by one session per week for 5 weeks, for a total of 10 sessions. The first five sessions were performed during hospitalization in our Pain Center. The permanent component of suprapubic pain was reduced from the second session (NRS: 6/10) and continued to gradually improve, to become well tolerated after the eighth session (NRS: 2/10) (Figure 1). The number of micturitions also decreased from the second session and was reduced by one half after the seventh session (between 12 and 13 micturitions per 24 h, including five nocturnal micturitions) (Figure 1). However, fatigue and sleep disorders persisted.

To treat depression, rTMS is usually applied either at high frequency (10–20 Hz) on the left DLPFC or at low frequency (1 Hz) on the right DLFPC. However, in one study, Speer et al. (2014) showed that rTMS of the left DLPFC, when applied at a high intensity of stimulation (110% of MT, as in our case), was similarly effective to produce long-term antidepressant effects whether it was delivered at low frequency (1 Hz) or high frequency (20 Hz), as usual. This surprising result led us to propose to our patient a series of monthly sessions of 1 Hz rTMS of the left DLPFC (1,200 pulses per session, delivered at 110% of MT) for 6 months. Permanent suprapubic pain, but also paroxysms of hypogastric sensation of pressure and urge to urinate completely vanished. Micturition frequency further decreased with an average of 10 micturitions per day (60% improvement compared to baseline) and two micturitions per night (80% improvement) (Figure 1). Sleep and fatigue similarly improved, with NRS scores of 3/10 (57% improvement) and 2/10 (60% improvement), respectively. The percentage of reduction of depression score was lower (30% improved, HAD score: 17/42). At the end of the series of 16 rTMS sessions, the patient was able to resume most of her activities of daily living with an overall satisfaction rate of 80%. It is planned to repeat sessions of 1 Hz rTMS of the left DLPFC two or three times a year in our Pain Center.

## Discussion

Bladder pain syndrome is an extremely disabling condition, including increased micturition frequency, urgency, nocturia, and chronic pain in the pelvic area, associated with depression, fatigue and sleep disorder, at the expense of the quality of life of patients (van de Merwe et al., 2008; Offiah et al., 2013; Hanno et al., 2015). Various hypotheses concerning BPS etiology and pathophysiology have been proposed, as mentioned in the introduction. Treatments can be invasive and expensive (Anger et al., 2011). For example, neuromodulation techniques, such as spinal cord stimulation (Pelaz et al., 2004) and especially sacral root stimulation (Srivastava, 2012), have shown some efficacy in patients with BPS refractory to other treatments. However, these techniques provide inconsistent results, more marked on pain than on urinary symptoms, and require surgical implantation of electrodes, in contrast to rTMS, which is non-invasive. In the context of pain therapy, rTMS is usually delivered on the motor cortex (Lefaucheur et al., 2011, 2014; Nizard et al., 2012). Good results have been reported in dysfunctional pain syndromes, such as fibromyalgia (Mhalla et al., 2011; Boyer et al., 2014), but not yet in irritable bowel syndrome (Melchior et al., 2014). Our case is the first reported experience of using focal high-frequency rTMS to alleviate BPS. Other techniques of rTMS can be applied for therapeutic purposes, using patterned stimulation settings, such as theta burst stimulation (Chung et al., 2015), or non-focal coils for large and deep brain stimulation, such as H-coils (Tendler et al., 2016). Very recently, one randomized sham-controlled crossover study evaluated the safety, tolerability, and efficacy of a protocol of 10 daily sessions of 20 Hz rTMS (1,500 pulses per session) using a H-coil positioned over the M1 area in a series of 13 patients with BPS (Cervigni et al., 2018). When compared with sham-rTMS, real-rTMS showed a significant efficacy on global pain intensity, lasting up to 6 weeks beyond the period of stimulation. The effects on lower urinary tract symptoms and quality of life were less impressive, and depression scores did not change. Conversely, in our observation, both pain and urinary tract symptoms improved in parallel, as illustrated in Figure 1. The quality of life was not specifically assessed, but the reduction from 10 to 2 nocturnal micturitions had a significant impact and therefore our patient also benefited from rTMS therapy regarding sleep and fatigue (57–60% improvement), and mood at a lower level (30% improvement).

First, it must be noticed that our patient developed BPS, while she was being efficaciously treated by spinal cord stimulation for pudendal neuralgia. This observation strongly suggests that these two clinical conditions do not result from the same central mechanisms, which is a very interesting and original observation.

Second, in terms of rTMS therapy for chronic pain syndromes, high-frequency rTMS of the motor cortex has the highest level of evidence of efficacy, at least regarding neuropathic pain, including at pelvic and perineal level (Louppe et al., 2013). However, according to our initial rTMS experience in the context of BPS, we performed low-frequency rTMS of the right DLPFC rather than high-frequency rTMS of the motor cortex. Since our patient was not sufficiently improved, we decided to stimulate the left DLPFC in addition to the right DLPFC. On the basis of a recent paper showing similar antidepressant efficacy of high and low frequency rTMS of the left DLPFC (Speer et al., 2014), rTMS was delivered to the left DLPFC at low frequency as on the right side. Our patient further improved, showing for the first time that a protocol of low-frequency rTMS of the DLPFC region of both hemispheres could be relevant in clinical practice. The efficacy of bihemispheric multi-target rTMS protocols had also been shown in Parkinson’s or Alzheimer’s disease (Lomarev et al., 2006; Nguyen et al., 2017).

The specific efficacy of DLPFC stimulation could indicate various mechanisms of action. First, antidepressant effects of rTMS of the DLPFC were found to involve functional connections of the DLPFC with the anterior cingulate cortex (ACC), including subgenual region (Fox et al., 2012; Liston et al., 2014; Philip et al., 2018). Descending pain modulation involves projections from the subgenual ACC to the periaqueductal gray (PAG) and the RVM (Calejesan et al., 2000; Kong et al., 2010; Cheng et al., 2015) and then to dorsal horn cells of the spine, but some connections from the ACC to spinal structures are able to modulate spinal sensory transmission in a RVM-independent manner (Aicher et al., 2012; Chen et al., 2018; Gomtsian et al., 2018).The RVM is also involved in the control of visceromotor reflexes to bladder distension dependent on the endogenous opioid system (Randich et al., 2008) and in modulating bladder pain: RVM neurons facilitate or inhibit the transmission of afferent sensory information from the bladder, including pain, and this is involved in the control of continence or micturition (Baez et al., 2005). In fact, the RVM neurons respond to unexpected stimuli of multiple modalities, noxious or innocuous, and are involved in behavioral adjustments due to salient situations unrelated to pain, such as the balance between micturition and continence, through multisynaptic projections to autonomic nervous structures (Mason, 2005a,b). Therefore, via the modulation of RVM activities in response to DLPFC stimulation by rTMS, urinary symptoms of BPS could be alleviated regardless of pain relief.

Another neural mechanism responsible for the analgesic effects of DLPFC stimulation may be related to the role played by the DLPFC in placebo analgesia (Schafer et al., 2018). Indeed, the possibility of a placebo effect cannot be excluded in this open-label case report. The DLPFC has repeatedly been shown to be involved in expectation-related placebo analgesia as well as in cognitive and attention-related regulation of pain (Mayberg et al., 2002; Benedetti et al., 2005; Wager and Atlas, 2015; Geuter et al., 2017), although the system is probably centered on the ventral part of the prefrontal cortex, e.g., in the context of visceral pain (Lu et al., 2010; Lee et al., 2012). During the anticipation of pain, the placebo effect was correlated with an increased activity in the DLPFC (Wager et al., 2004) and this triggers brain activation in a large network of limbic and brainstem structures involved in pain regulation. These structures, such as the RVM or the PAG, are especially involved in endogenous descending modulatory controls involved in placebo inhibition in a wide range of painful experiences (Eippert et al., 2009; Büchel et al., 2014; Wager and Atlas, 2015; Koban et al., 2017). However, in healthy subjects, one previous study showed that a succession of two stimulation trains of 1Hz-rTMS for 15 min, delivered on the left then on the right DLPFC was able to block expectation-related placebo analgesia (Krummenacher et al., 2010). Thus, this observation argues against a placebo effect at the origin of our results, even if we applied low-frequency rTMS at different time on the right and left DLPFC and not in the same session. Also, against a placebo effect, it must be emphasized that our patient was refractory to various drug or invasive treatments (e.g., hydrodistention) before being treated by rTMS.

This first case of BPS treatment by low frequency rTMS applied to the DLPFC of both hemispheres paves the way for further studies. In the context of BPS, a clinical condition difficult to manage, non-invasive cortical stimulation should be considered as a therapeutic option, before attempting more invasive treatments such as cystectomy (Anger et al., 2011).

## Author Contributions

JN, JE, BB, AS, J-PL, and J-PN made substantial contributions to the conception and design of the work, and the acquisition, analysis, and interpretation of data for the work. JN, J-PL, and J-PN drafted the work and revised it critically for important intellectual content.

## Conflict of Interest Statement

The authors declare that the research was conducted in the absence of any commercial or financial relationships that could be construed as a potential conflict of interest.
